# Global Snow-free Leaf Area Index Dataset 1985–2020 for Earth System Modeling

**DOI:** 10.1038/s41597-026-07677-3

**Published:** 2026-06-29

**Authors:** Wanyi Lin, Hua Yuan, Wenzong Dong, Zhuo Liu, Jiayi Xiang, Yongjiu Dai

**Affiliations:** 1 School of Atmospheric Sciences, Sun Yat-sen University, Zhuhai, 519082 China; 2 Guangzhou Meteorological Satellite Ground Station (Guangdong Meteorological Satellite Remote Sensing Centre), Guangzhou, 510640 China

## Abstract

Leaf Area Index (LAI) is a fundamental parameter linking vegetation structure with surface energy and carbon exchange in Earth system models. However, the underestimation of LAI caused by snow cover remains a persistent limitation of existing satellite products. Here, we develop a global snow-free LAI dataset covering the years 1985–2020 at 500 m resolution. The method compiles over 2700 leaf lifespan records from the TRY plant trait database, spanning observations for numerous plant species, and aggregates them into plant functional type (PFT)-specific values. It then identifies snow-affected regions using MODIS data and applies a leaf-lifespan-based correction to PFT-specific LAI values. The physiologically constrained snow-free LAI effectively corrects the underestimation of LAI in snow-affected regions. Simulations with the Common Land Model indicate that snow-free LAI improves albedo simulations over snow-covered regions by better representing vegetation masking effects and reducing positive albedo bias. Additionally, the snow-free LAI increases net radiation and gross primary productivity, and reduces snow depth. This snow-free LAI dataset provides a reliable input for modeling vegetation-snow interactions in Earth system models, supporting more accurate simulations of surface energy, water and carbon dynamics.

## Background & Summary

Leaf Area Index (LAI) serves as a key parameter linking vegetation canopy structure with land-atmosphere interactions. It fundamentally affects the simulation of radiation transfer, precipitation interception, and biogeochemical processes in land surface and climate models. Compared with site-based observations that are limited in spatial coverage and temporal frequency, remote sensing–retrieved LAI data provide valuable information from regional to global scales and from intra-monthly to interannual variations. Since the 1980s, a series of global LAI products with varying spatial and temporal resolutions have been developed. These include products derived from the Moderate Resolution Imaging Spectroradiometer (MODIS)^1–3^, the Global Land Surface Satellite (GLASS) LAI product^4^, the Sensor-independent Leaf Area Index/Fraction of Photosynthetically Active Radiation Climate Data Record (SI LAI/FPAR CDR)^5^, the Global Inventory Modeling and Mapping Studies (GIMMS) LAI4g product^6^, the High-Quality LAI (HiQ-LAI)^7^, and the Advanced Very High Resolution Radiometer (AVHRR)-derived AVH15C1 product^8^, among others. However, remotely sensed LAI products are still subject to uncertainties caused by clouds, snow, and aerosols, as well as sensor malfunctions, variations in solar–target–sensor geometry, and algorithmic uncertainties, among other factors. These factors often result in data gaps and noise, limiting the applicability of such products in model simulations and data analyses^9–12^. Although several of the aforementioned datasets have undergone reprocessing and optimization, snow-related biases persist, particularly during winter periods.

As many studies have highlighted, the underestimation of LAI caused by snow cover on the canopy is a widely recognized issue^13–16^. In high-latitude regions during winter, remote sensing-retrieved LAI is hampered by low solar elevation angles and the presence of snow cover. Uncertainties in LAI propagate through multiple pathways, thereby influencing key variables in land surface modeling such as surface albedo, snow water equivalent, snowmelt runoff, and soil moisture^17–19^. In boreal coniferous forests, underestimated LAI can result in overestimation of surface albedo, which in turn leads to underestimation of surface temperature^20,21^. Moreover, previous research has indicated that discrepancies among global climate models in simulating albedo and snow-albedo feedback largely stem from differing treatments of vegetation-snow interactions^22^. Therefore, accurately representing snow-free LAI is critical for improving albedo simulations and reducing inter-model uncertainty in land surface and climate models^23^. LAI also influences other key land surface processes by regulating canopy radiative transfer, thereby controlling the absorption of incoming radiation and increasing net radiation, which in turn enhances gross primary productivity (GPP) by increasing the absorption of photosynthetically active radiation^23,24^. In addition, vegetation structure plays a critical role in snow interception, as denser canopies can intercept more snowfall and reduce the amount of snow reaching the ground, thereby affecting snow accumulation and snow depth^25^.

Few studies have addressed the impact of snow on LAI products at the global scale. Even regional approaches using airborne or Unmanned Aerial Vehicle (UAV)-based LAI observations have not resolved snow-related biases^26,27^. To incorporate remotely sensed LAI into models, some studies have applied forced adjustments to winter LAI for evergreen plant functional types (PFTs)^28,29^, ensuring that the plant functional type (PFT)-specific LAI (PFT LAI) of evergreen needleleaf trees remains above 70% of its annual maximum. Park *et al*.^30^ incorporated a LAI dataset derived from a global synthesis of more than 1,000 published estimates^31^ into the Noah-MP model and found that simulated albedo performance was significantly improved compared with that using a fixed minimum LAI parameter. Wang *et al*.^17^ applied LAI corrections to remotely sensed time series based on leaf lifespan data of evergreen coniferous species, and demonstrated improvements in albedo simulation. Although this approach was limited to the regional scale and species level, it provides a practical and transferable framework for correcting snow-affected LAI. Nevertheless, a globally consistent, high-resolution LAI dataset that adequately addresses winter underestimation remains lacking for land surface and climate modeling applications.

Therefore, this study aims to develop a global snow-free LAI dataset (1985–2020) based on long-term LAI products, including the GIMMS LAI4g product^6^ and the reprocessed MODIS LAI products^32^, which were already harmonized within the LAI4g framework to ensure temporal consistency, enabling its effective application in land surface models. We extend the correction method proposed by Wang *et al*.^17^ by incorporating over 2700 site-level leaf lifespan records and satellite-derived snow extent information, thereby enabling the generation of a globally consistent, snow-free LAI dataset. The extended approach involves (1) translation of species-level leaf lifespan data into PFT categories commonly used in land surface models; (2) application of MODIS quality control (QC) information to identify snow-covered areas requiring correction; and (3) LAI correction performed at the PFT level using a newly developed PFT-specific LAI product, thereby resolving the scale mismatch between species-level leaf lifespan information and grid-scale LAI products. Subsequently, the snow-free LAI is derived using PFT fractional cover as weights. This procedure effectively reduces spurious seasonal variability in evergreen-dominated regions and yields temporally stable LAI consistent with evergreen phenology. The effectiveness of the snow-free LAI was assessed in the Common Land Model version 2024 (CoLM 2024) through both site-scale and regional-scale simulations. The results indicate that correcting snow-season LAI effectively reduces the overestimation of winter albedo when using the reprocessed LAI data. This improvement arises from enhanced canopy masking of snow, which reduces the contribution of snow-covered background surfaces to the total albedo. Beyond albedo, the snow-free LAI induces a physically consistent land surface response, including increased net radiation, enhanced GPP, and reduced snow depth through stronger canopy interception. These changes highlight the important role of realistic canopy structure in regulating surface energy balance and snow-vegetation interactions. Overall, the snow-free LAI provides more reliable LAI estimates and thereby supports simulations of energy, water and carbon fluxes in land surface and climate models.

## Methods

The 500 m PFT data used in this study were obtained by aggregating the 30 m PFT distribution dataset (PFT30)^33^. The PFT30 dataset was developed by integrating multiple high-resolution remote sensing products and climate background datasets to derive fractional PFT cover, following a classification scheme that distinguishes major vegetation types and their phenological characteristics. The dataset spans 1985–2020 and is temporally varying, with updates at five-year intervals before 2000 and annually thereafter. It has been evaluated against site-level observations and compared with other global PFT products, with results reported in Lin *et al*.^33^.

The 500 m PFT LAI data^33^ used in this study were derived from a previously developed product, which integrates the reprocessed MODIS LAI product^32^, the GIMMS LAI4g product^6^, and the PFT30 product through a remote-sensing-based phenology scheme. Specifically, LAI values for 1985–2003 were derived from the GIMMS LAI4g dataset; values from 2004 onward were obtained from the reprocessed MODIS LAI product. It should be noted that this is not a simple combination of independent datasets, as LAI4g has been explicitly harmonized with the reprocessed MODIS LAI through pixel-wise fusion during the overlapping period, ensuring spatiotemporal consistency across the AVHRR and MODIS eras. Based on this consistent LAI input, the method links grid LAI with dominant PFT information to estimate PFT LAI and subsequently partitions LAI among PFTs at 500 m resolution. This enables the PFT LAI product to capture realistic phenological variations by leveraging remote sensing information, thereby overcoming limitations of traditional empirical approaches.

It should be noted that the datasets described above correspond to our recent preprint in Earth System Science Data Discussions (ESSDD)^33^, which details the generation of PFT LAI from grid-scale satellite observations. In contrast, the present study does not aim to further develop the PFT partitioning framework, but instead addresses a different and independent issue, namely the systematic bias in LAI under snow conditions. The correction introduced here is therefore a distinct methodological extension that operates on the previously derived dataset and involves substantial processing of site-based plant trait data, rather than a re-presentation of the same data product.

Building on these datasets, the present study first corrected the PFT LAI data of evergreen PFTs using the average leaf lifespan of each PFT. The PFT classification in PFT30 includes five evergreen types: Temperate Needleleaf Evergreen Tree (TeNET), Boreal Needleleaf Evergreen Tree (BoNET), Tropical Broadleaf Evergreen Tree (TrBET), Temperate Broadleaf Evergreen Tree (TeBET) and Temperate Broadleaf Evergreen Shrub (TeBES). The corrected PFT LAI was then aggregated to update grid-scale LAI through area-weighted averaging based on PFT fractional cover derived from the PFT30 dataset. Consequently, the final LAI product provides both PFT LAI and grid LAI values.

The average leaf lifespan of PFTs was derived based on the TRY plant trait database^34^. TRY is a comprehensive and widely used plant trait database that compiles measurements across species, growth forms, life stages, and environmental conditions. Species-level datasets, including leaf lifespan, PFT classification, growth form, and species-trait lookup tables, were extracted from the TRY database and used as ancillary information to derive representative average leaf lifespans for each PFT.

The snow-detected flag indicating the presence of snow cover was obtained from the FparExtra_QC layer of the MODIS MCD15A2H C6.1 LAI product^35^. This flag is closely linked to the reliability of LAI retrievals and thus shows strong consistency with the underlying remote sensing observations. It was used to identify snow-affected grid cells, defined as those experiencing snow cover at any time during the year, and the correction was applied to PFT LAI.

For the correction, a consistent adjustment was applied to snow-affected grid cells across the entire annual cycle, rather than applying seasonally varying adjustments. A representative year (2005) was used for the snow flag, as the method only requires identifying whether a grid cell experiences snow cover at any time, rather than the exact timing or frequency of snow events. This approach ensures temporal continuity and avoids artificial fluctuations between corrected and uncorrected periods. An alternative strategy based on temporally varying snow-detected flags was tested, but it introduced unrealistic discontinuities in the LAI time series (results not shown). Therefore, the correction method is effectively insensitive to snow season duration and reduces sensitivity to potential misclassification caused by cloud cover or aerosol contamination.

The principle of PFT LAI correction using leaf lifespan is based on the fact that leaf lifespan reflects the average residence time of leaves on a plant. The reciprocal of leaf lifespan, i.e., the leaf turnover rate, represents the proportion of leaves shed within a year. This allows the determination of the minimum proportion of leaves that should be retained throughout the year, expressed as follows:

1 $${{LAI}}_{\min }^{{\prime} }=(1-1/\tau ){{LAI}}_{{RS},\max }$$

where *τ* represents leaf lifespan (unit: year), 1−1/τ represents the annual leaf retention fraction, ${{LAI}}_{{RS},\max }$ is the annual maximum PFT LAI, and ${{LAI}}_{\min }^{{\prime} }$ is the annual minimum PFT LAI constrained by leaf lifespan. Note that ${{LAI}}_{\min }^{{\prime} }$ is additionally bounded below by the satellite-observed annual minimum LAI, ensuring that the correction does not reduce LAI below values already present in the remote sensing record. According to this expression, when τ=1, ${{LAI}}_{\min }^{{\prime} }$=0, indicating that leaves may be completely shed within a year, corresponding to deciduous species. For τ > 1, a larger τ indicates longer leaf residence time and lower leaf turnover, resulting in a larger ${{LAI}}_{\min }^{{\prime} }$.

Although leaf lifespan is measured at the species level, globally complete species-level distribution data at high spatial resolution are currently unavailable. Directly mapping species-specific leaf lifespan to each grid cell would therefore introduce substantial data gaps and uncertainties. To overcome this limitation, species-level leaf lifespan was aggregated to plant functional types, which represent the functional vegetation classes used in land surface models.

**Fig. 1:**
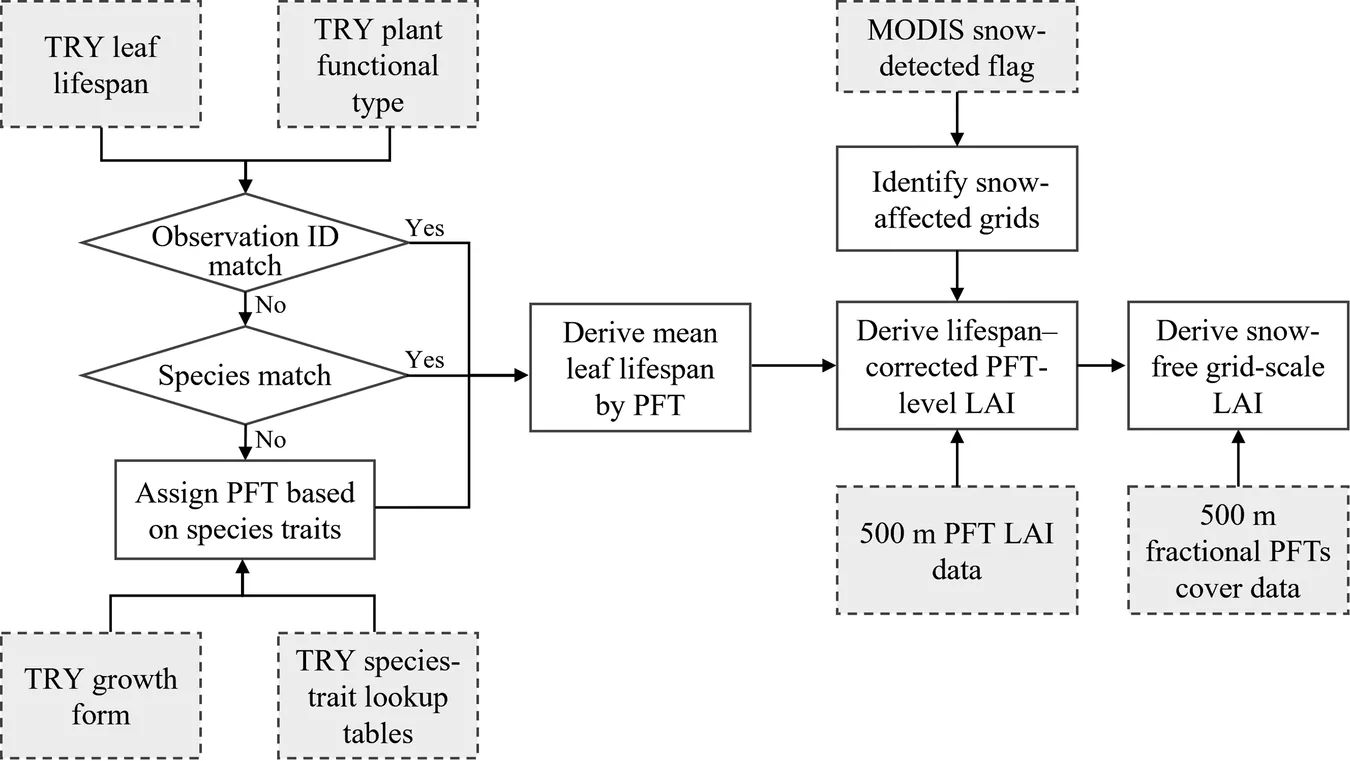
Workflow for deriving PFT-specific mean leaf lifespan and generating snow-free LAI.

To upscale species-scale leaf lifespan to the PFT scale, the average leaf lifespan for each PFT was derived through a hierarchical classification procedure (Fig. 1). First, records in the leaf lifespan database were matched with the PFT database using observation identifiers. Second, for leaf lifespan records that could not be matched by observation ID, the corresponding PFT type was determined based on species names, provided that the species existed in the PFT database. When no match could be established through either observation ID or species name, the PFT type of the species in the leaf lifespan database was inferred using ancillary information retrieved from the TRY plant trait database, including growth form data and species–trait lookup tables. The lookup tables provide information on phenology, leaf type, and photosynthetic pathway, which, in combination with growth form data, enable species-level PFT classification. Finally, for a small number of remaining species that could not be classified through the above steps, supplementary information from online botanical resources was consulted as supporting information to complete the PFT assignment.

Based on the resulting species-PFT mapping, species-level leaf lifespan observations were then aggregated to obtain representative average lifespans for each PFT. To ensure the reliability of the above processing steps, leaf lifespan data were quality-controlled following TRY database guidelines, and species with ambiguous PFT characteristics were classified based on leaf lifespan and growth form criteria, thereby ensuring consistency in PFT assignment.

**Fig. 2:**
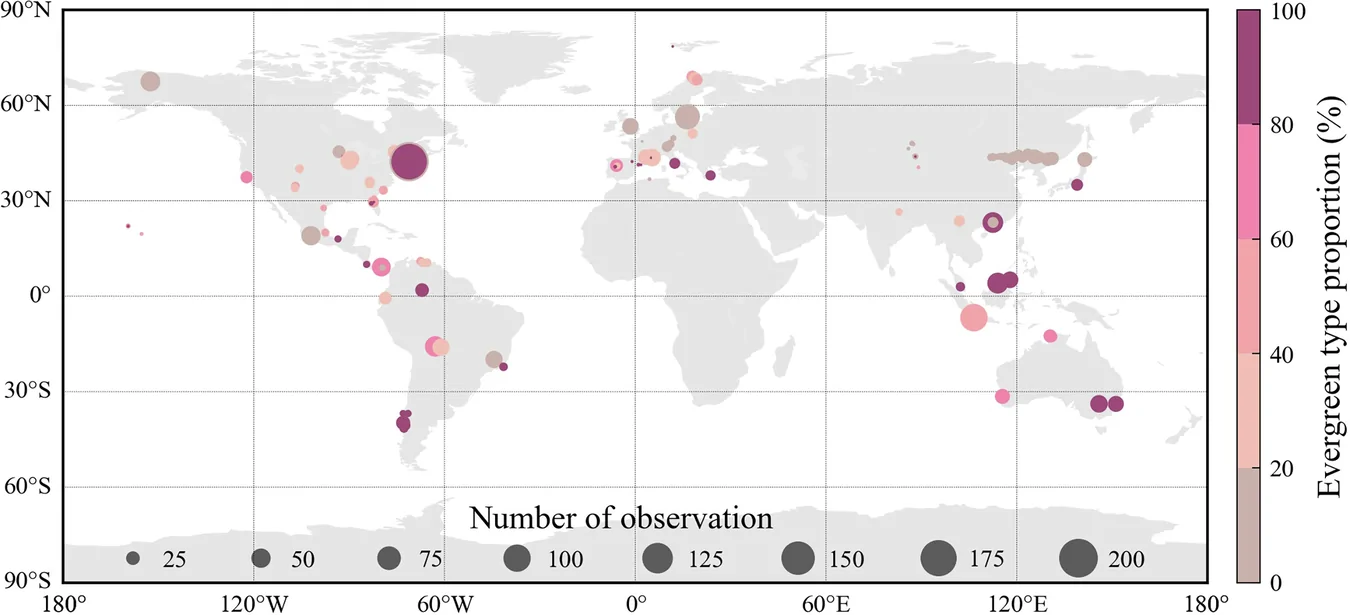
Spatial distribution of leaf lifespan observation sites. Circle size represents the number of observations at each site, and color indicates the proportion of observations from evergreen species.

**Fig. 3:**
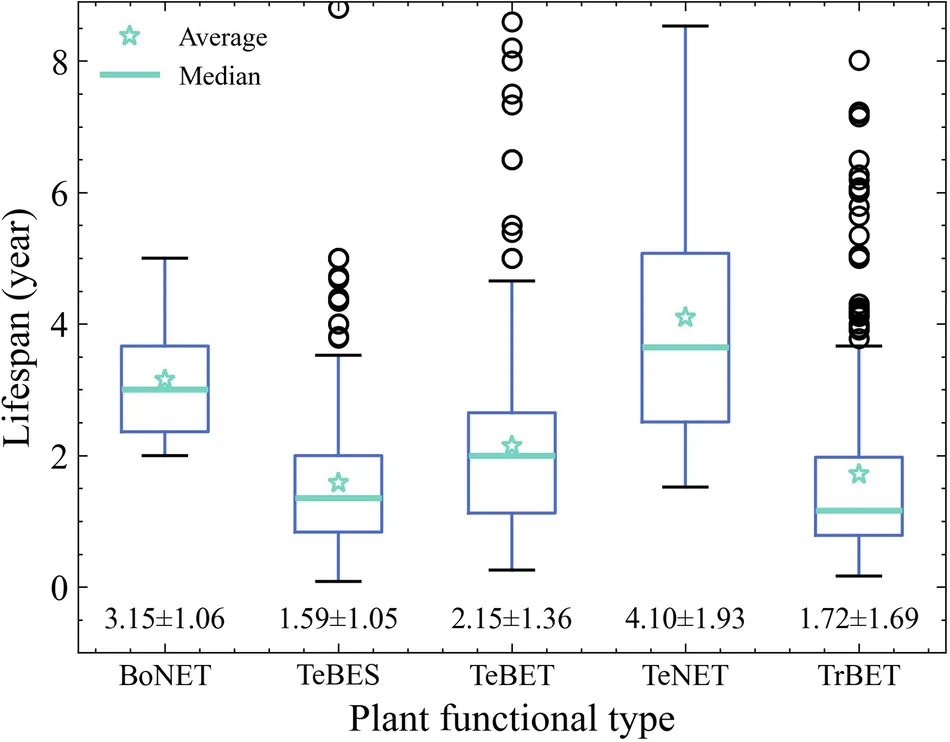
Leaf lifespan distribution of evergreen PFTs based on site-level observations, with mean ± standard deviation annotated to quantify within-PFT variability.

The resulting dataset comprises 2,762 valid leaf lifespan records with corresponding PFT information from 1,630 species (Fig. 2), with values ranging from 0.02 to 14.58 years. Among these, 1,275 observations correspond to evergreen PFTs. Across the five evergreen PFTs, TrBET exhibits the shortest leaf lifespan, whereas TeNET shows the longest mean lifespan (4.1 years) and the greatest data dispersion (Fig. 3). As the dominant vegetation in snow-covered regions, BoNET has a mean leaf lifespan of 3.15 years and shows the lowest dispersion among the four evergreen tree PFTs, indicating that the uncertainty in snow-free LAI data is expected to be limited.

Using the average leaf lifespan derived above, the PFT LAI in snow-affected grids was adjusted according to the following equation:

2 $${{LAI}}^{t}={{LAI}}_{\min }^{{\prime} }+\frac{{{LAI}}_{{RS}}^{t}-{{LAI}}_{{RS},\min }}{{{LAI}}_{{RS},\max }-{{LAI}}_{{RS},\min }}({{LAI}}_{{RS},\max }-{{LAI}}_{\min }^{{\prime} })$$

where ${{LAI}}^{t}$ is the snow-free PFT LAI at time step t, ${{LAI}}_{\min }^{{\prime} }$ is the yearly minimum PFT LAI estimated from leaf lifespan, ${{LAI}}_{{RS},\max }$ and ${{LAI}}_{{RS},\min }\,$ are the annual maximum and minimum PFT LAI values derived from integrated remote-sensing products, including the reprocessed LAI and PFT30 datasets, and ${{LAI}}_{{RS}}^{t}$ is the PFT LAI at time step t. The snow-free PFT LAI time series were then aggregated to the grid scale using fractional PFT cover as weights, producing the grid snow-free LAI dataset. The workflow for deriving PFT-specific mean leaf lifespan and applying the LAI correction is illustrated in Fig. 1.

## Data Records

The dataset is available at Zenodo (https://doi.org/10.5281/zenodo.18098037)^36^. It provides monthly LAI and corresponding Stem Area Index (SAI) at 500 m spatial resolution globally, distributed as NetCDF-4 format files (Table 1). Each file covers a 5° × 5° geographic region containing 1200 × 1200 pixels (0.00416667° or 15 arc-seconds per pixel) in the WGS84 coordinate system. The global land surface is divided into 1,447 regional tiles, with files named according to the convention RG_LatN_LonW_LatS_LonE.LSAI500m.YYYY.nc, where the latitude and longitude bounds define the tile coverage and YYYY indicates the year.

**Table 1:** Summary of the dataset properties.

Attribute	Description
Temporal Resolution and Coverage	Monthly (12 months per year), 1985–2020
Spatial Resolution and Coverage	500 m (0.00416667°, 15 arc-seconds), 5° × 5° per tile, 1,447 tiles covering global land areas
Coordinate Reference System	Geographic coordinate system (WGS84), degrees longitude and latitude
File Format	NetCDF-4
Naming Convention	RG_LatN_LonW_LatS_LonE.LSAI500m.YYYY.nc where LatN/LatS are northern/southern latitude bounds, LonW/LonE are western/eastern longitude bounds, and YYYY stands for year
Data Valid Range and Scale Factor	0–100, scale factor 0.1
Data Volume	~30 GB per year for 1985–2003, ~40 GB per year for 2004–2020

Due to the large volume of the complete long-term dataset, annual datasets are archived separately under individual Zenodo repositories. The corresponding DOIs for each year are listed below:

Year 1985: https://doi.org/10.5281/zenodo.19973570^37^

Year 1986: https://doi.org/10.5281/zenodo.20022238^38^

Year 1987: https://doi.org/10.5281/zenodo.20022240^39^

Year 1988: https://doi.org/10.5281/zenodo.20022401^40^

Year 1989: https://doi.org/10.5281/zenodo.20033581^41^

Year 1990: https://doi.org/10.5281/zenodo.20022460^42^

Year 1991: https://doi.org/10.5281/zenodo.20033663^43^

Year 1992: https://doi.org/10.5281/zenodo.20033691^44^

Year 1993: https://doi.org/10.5281/zenodo.20033684^45^

Year 1994: https://doi.org/10.5281/zenodo.20049108^46^

Year 1995: https://doi.org/10.5281/zenodo.20022480^47^

Year 1996: https://doi.org/10.5281/zenodo.20049104^48^

Year 1997: https://doi.org/10.5281/zenodo.20049210^49^

Year 1998: https://doi.org/10.5281/zenodo.20049212^50^

Year 1999: https://doi.org/10.5281/zenodo.20065802^51^

Year 2000: https://doi.org/10.5281/zenodo.19729874^52^

Year 2001: https://doi.org/10.5281/zenodo.19736095^53^

Year 2002: https://doi.org/10.5281/zenodo.19759697^54^

Year 2003: https://doi.org/10.5281/zenodo.19772792^55^

Year 2004: https://doi.org/10.5281/zenodo.19782009^56^

Year 2005: https://doi.org/10.5281/zenodo.18098037^36^

Year 2006: https://doi.org/10.5281/zenodo.19782092^57^

Year 2007: https://doi.org/10.5281/zenodo.19784933^58^

Year 2008: https://doi.org/10.5281/zenodo.19804507^59^

Year 2009: https://doi.org/10.5281/zenodo.19804804^60^

Year 2010: https://doi.org/10.5281/zenodo.19805222^61^

Year 2011: https://doi.org/10.5281/zenodo.19827037^62^

Year 2012: https://doi.org/10.5281/zenodo.19839997^63^

Year 2013: https://doi.org/10.5281/zenodo.19839968^64^

Year 2014: https://doi.org/10.5281/zenodo.19840551^65^

Year 2015: https://doi.org/10.5281/zenodo.18130709^66^

Year 2016: https://doi.org/10.5281/zenodo.19883407^67^

Year 2017: https://doi.org/10.5281/zenodo.19910424^68^

Year 2018: https://doi.org/10.5281/zenodo.19910449^69^

Year 2019: https://doi.org/10.5281/zenodo.19910541^70^

Year 2020: https://doi.org/10.5281/zenodo.19943218^71^

Each file contains four primary data variables: MONTHLY_LC_LAI and MONTHLY_LC_SAI representing 500 m grid LAI and SAI values with dimensions (12 months × 1200 lat × 1200 lon), and MONTHLY_PFT_LAI and MONTHLY_PFT_SAI providing PFT LAI and SAI values with dimensions (12 months × 16 PFTs × 1200 lat × 1200 lon). Grid LAI (SAI) and PFT LAI (SAI) are both available for user selection according to research demands. The grid LAI (SAI) is calculated as the area-weighted average of PFT LAI (SAI).

All LAI and SAI values are stored as unsigned byte integers with a scale factor of 0.1 and units of m^2^/m^2^, requiring multiplication by 0.1 to obtain physical values. The 16 plant functional types include various tree, shrub, grass, and crop categories ranging from tropical to boreal biomes. Coordinate variables (lat, lon, pft, mon) are included with appropriate metadata.

## Technical Validation

**Fig. 4:**
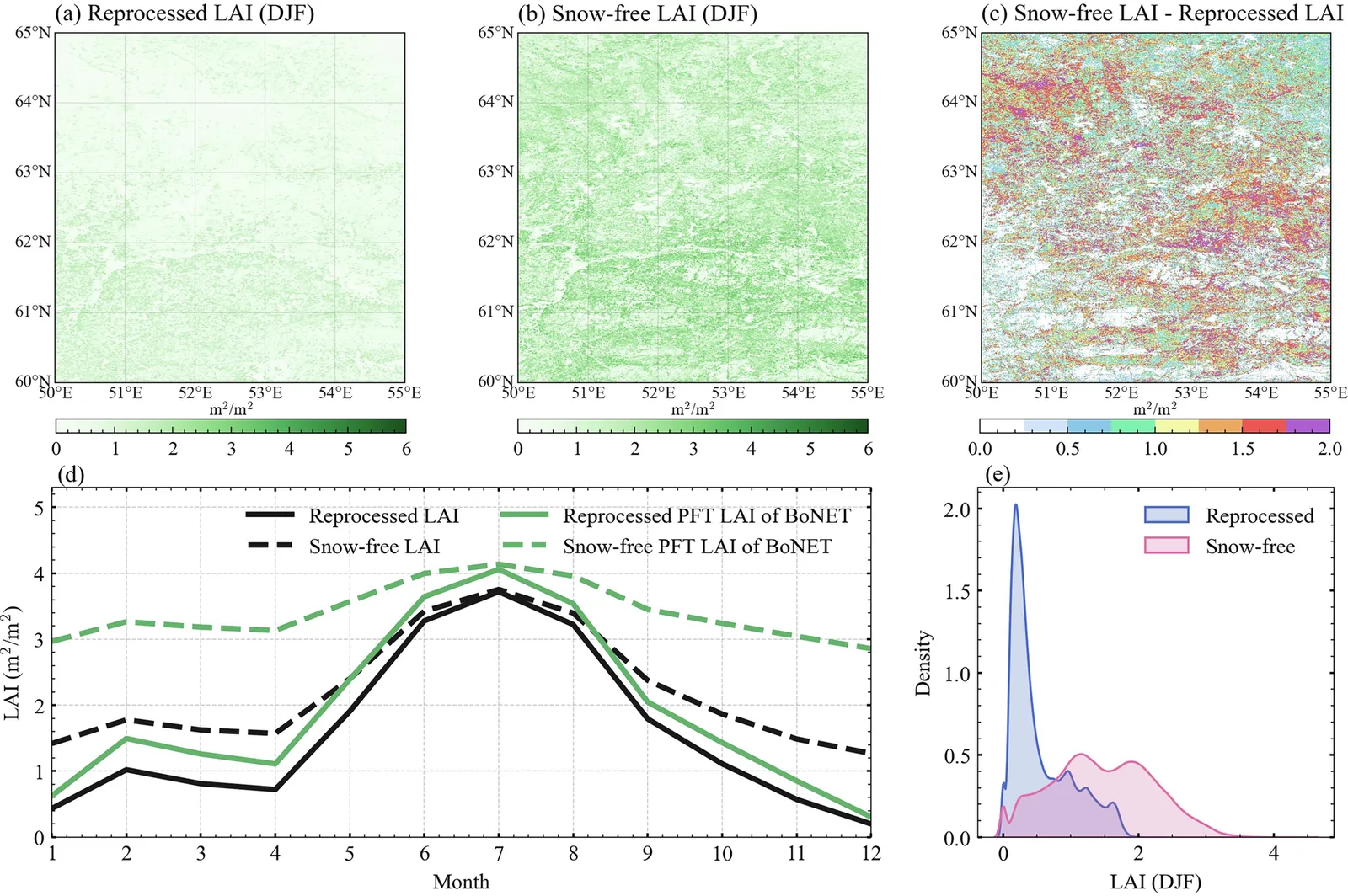
Comparison of LAI before and after leaf-lifespan-based correction in the 60–65°N, 50–55°E region at 500 m resolution in 2005: (**a**) Reprocessed LAI; (**b**) Snow-free LAI; (**c**) LAI difference (Snow-free LAI ₋ Reprocessed LAI); (**d**) Time series of regional mean grid LAI (black) and BoNET PFT LAI (green) before and after correction; (**e**) Distribution of grid LAI values before and after correction.

We selected a 5° × 5° region (60–65°N, 50–55°E) with extensive evergreen vegetation at 500 m resolution to evaluate LAI changes before and after correction. Approximately 40% of the grid cells are dominated by BoNET, defined as cells with a BoNET fractional cover greater than 50%. As shown in Fig. 4, winter values of the reprocessed LAI were close to zero across most of the region due to strong snow cover influence, whereas the snow-free LAI increased notably after being constrained by leaf lifespan data. Time series of PFT-level and grid-scale LAI indicate that the peak LAI in July remained unchanged after correction, while values in other months, particularly during winter, increased (Fig. 4d). The grid-scale increase is smaller than the PFT-level increase because about 22% of the area is covered by deciduous trees and 27% by deciduous shrubs, for which no adjustment was applied. Before correction, LAI values were mostly within the 0 to 1 m^2^/m^2^ range (Fig. 4e), whereas after correction, the proportion of grid cells with LAI exceeding 2 m^2^/m^2^ increased significantly.

**Fig. 5:**
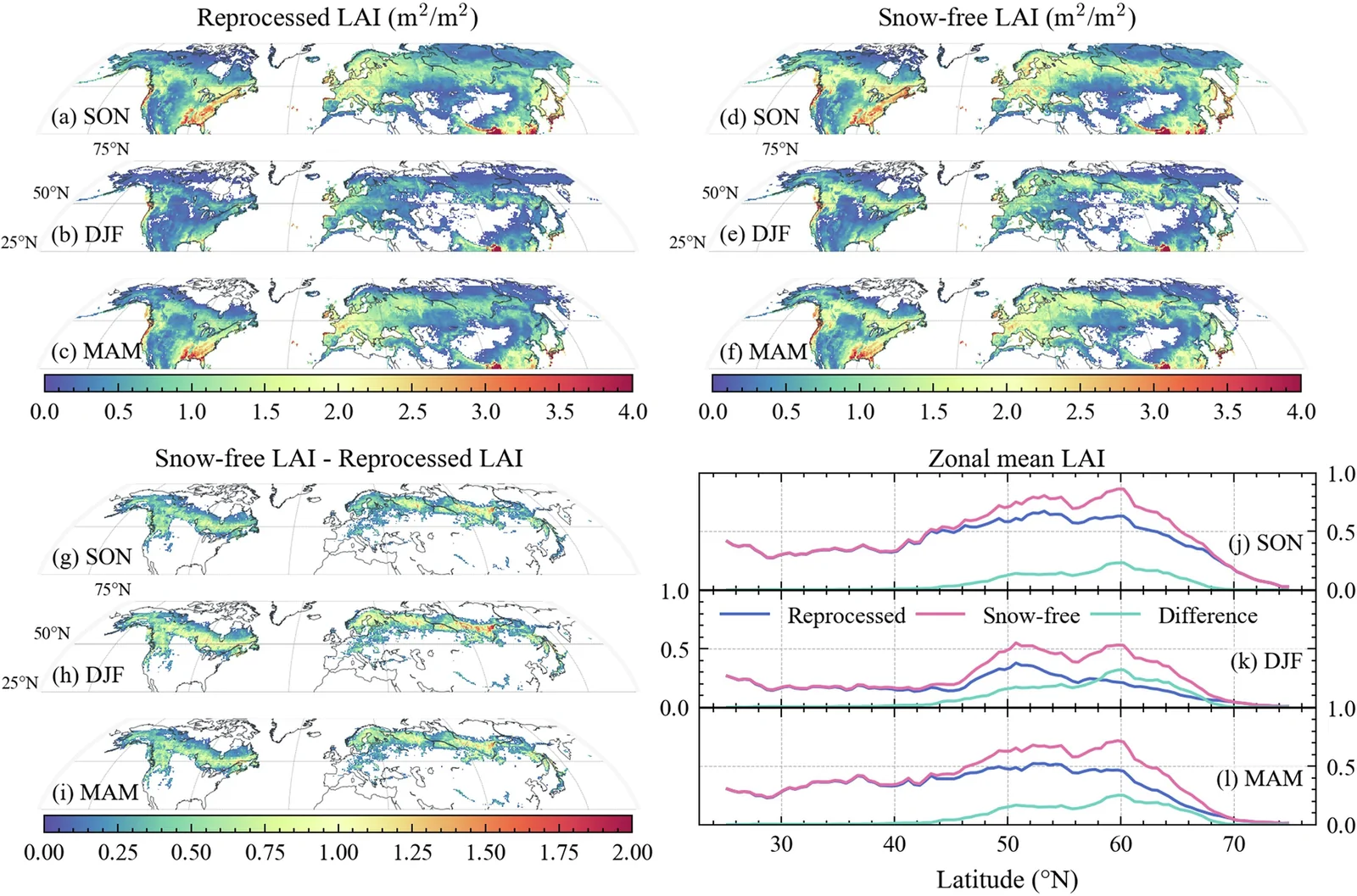
Comparison of seasonal grid-scale LAI at 0.5° resolution before and after correction in 2005. (**a**–**c**) Reprocessed LAI for autumn (September–October–November, SON), winter (December–January–February, DJF), and spring (March–April–May, MAM), respectively; (**d**–**f**) snow-free LAI for the same seasons. Grids with LAI less than 0.1 m^2^/m^2^ are masked and shown in white; (**g**–**i**) LAI difference between reprocessed and snow-free LAI; (**j**–**l**) zonal mean reprocessed LAI and snow-free LAI, together with their differences.

For the global-scale leaf-lifespan-based correction, Fig. 5 shows LAI variation at 0.5° resolution in snow-affected regions in 2005. Prior to correction, the reprocessed LAI exhibits pronounced seasonal fluctuations (Fig. 5a–c), particularly in BoNET-dominated grids (fractional cover >50%), where LAI decreases from 1.36 m^2^/m^2^ in autumn to 0.75 m^2^/m^2^ in winter before gradually rising to 1.10 m^2^/m^2^ in spring, inconsistent with the evergreen phenology. After applying the leaf-lifespan-based correction to evergreen PFTs, these seasonal discrepancies were substantially reduced (Fig. 5d–f), with mean LAI in BoNET-dominated areas reaching 2.19, 1.81, and 2.00 m^2^/m^2^ in autumn, winter, and spring, respectively. Spatially, LAI increases were concentrated in BoNET-dominated regions roughly between 40° N and 70° N, where winter LAI increased by an average of 1.06 m^2^/m^2^ (Fig. 5g–i). Zonal mean comparison (Fig. 5j–l) further highlights the seasonal pattern, with the largest enhancement in winter (0.32 m^2^/m^2^ at 60° N), and smaller but comparable increases in spring (0.23 m^2^/m^2^) and autumn (0.25 m^2^/m^2^). The overall zonal mean increase is lower than the increase in BoNET-dominated grids, due to the presence of non-evergreen PFTs such as deciduous shrubs, grasses, and crops.

**Fig. 6:**
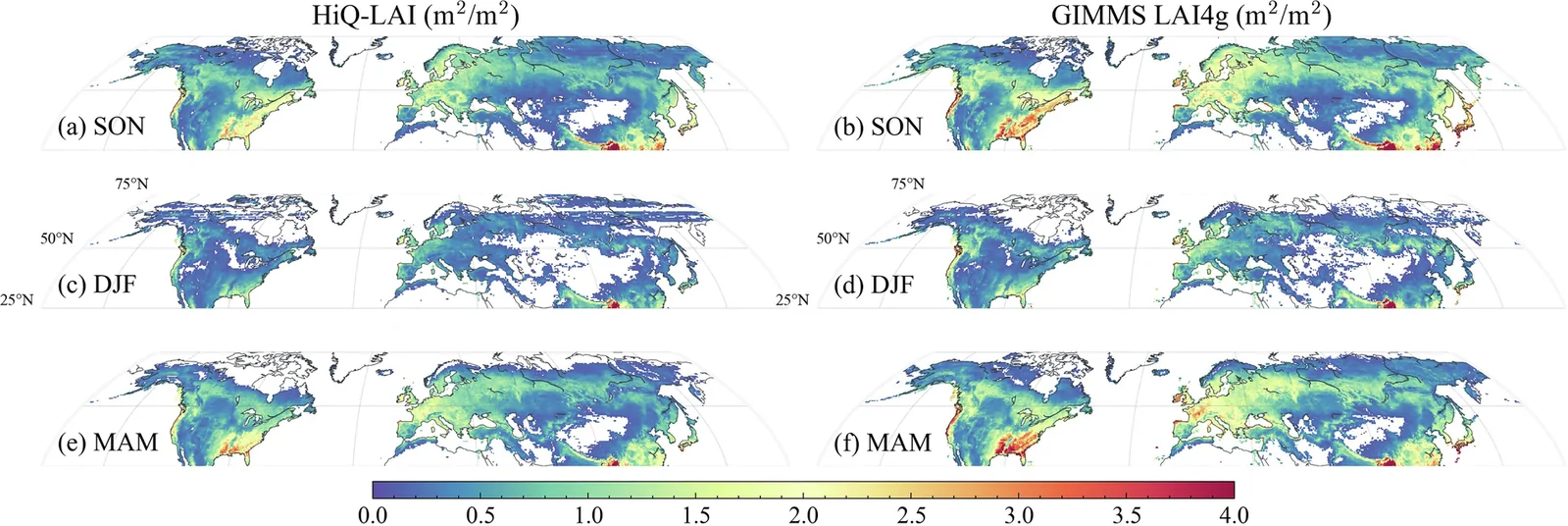
Seasonal LAI distributions at 0.5° resolution in 2005 for HiQ-LAI (left column) and LAI4g (right column) in autumn (SON; a, b), winter (DJF; c, d), and spring (MAM; e, f). Grids with LAI less than 0.1 m^2^/m^2^ are masked and shown in white. For HiQ-LAI, an additional quality filter was applied: grid cells where fewer than 50% of the constituent 5 km pixels contained valid retrievals within a given month were excluded to reduce noise from sparse sampling under heavy cloud or low solar elevation conditions.

To examine whether the observed snow-season LAI depression is consistent across datasets, we compared the reprocessed LAI with two other satellite-derived LAI products, HiQ-LAI and LAI4g (Fig. 6). Both products show a significant winter reduction in boreal forest regions similar to that found in the reprocessed LAI, suggesting that snow contamination is a systematic issue affecting optical remote-sensing LAI retrievals. In boreal forest regions, both HiQ-LAI and LAI4g yield winter LAI values around or below 1.0 m^2^/m^2^, consistent with the pre-correction reprocessed LAI. These cross-dataset comparisons support the interpretation that snow-season LAI suppression in boreal forests is a pervasive snow-related artifact, highlighting the necessity of the proposed leaf-lifespan-based correction.

The impact of the snow-free LAI data on land surface modeling was evaluated in the Common Land Model version 2024 (CoLM 2024)^72^ through both site-scale and regional-scale simulations. CoLM 2024 incorporates an explicit vegetation–snow interaction scheme that represents snow interception by the canopy and modulates surface albedo variations induced by snow cover. Simulations were conducted using the Plant Community (PC)-based subgrid scheme^73^, which accounts for the three-dimensional structure of vegetation canopies. A nine-year spin-up period was performed by repeating the 1998–2000 forcing data three times to reach equilibrium, followed by a continuous simulation from 2001 to 2010 for evaluation and analysis. Key model schemes and configuration settings are summarized in Table 2.

**Table 2:** Summary of model configuration for site-scale and regional-scale simulations.

	Description
Model	CoLM 2024
Subgrid scheme	Plant Community (PC)-based scheme
Snow scheme	Explicit vegetation-snow interaction scheme
Spin-up	9 years (1998–2000 repeated)
Simulation period	2001–2010
Experiments	Exp_RSLAI (reprocessed LAI as input)
	Exp_SFLAI (snow-free LAI as input)

**Fig. 7:**
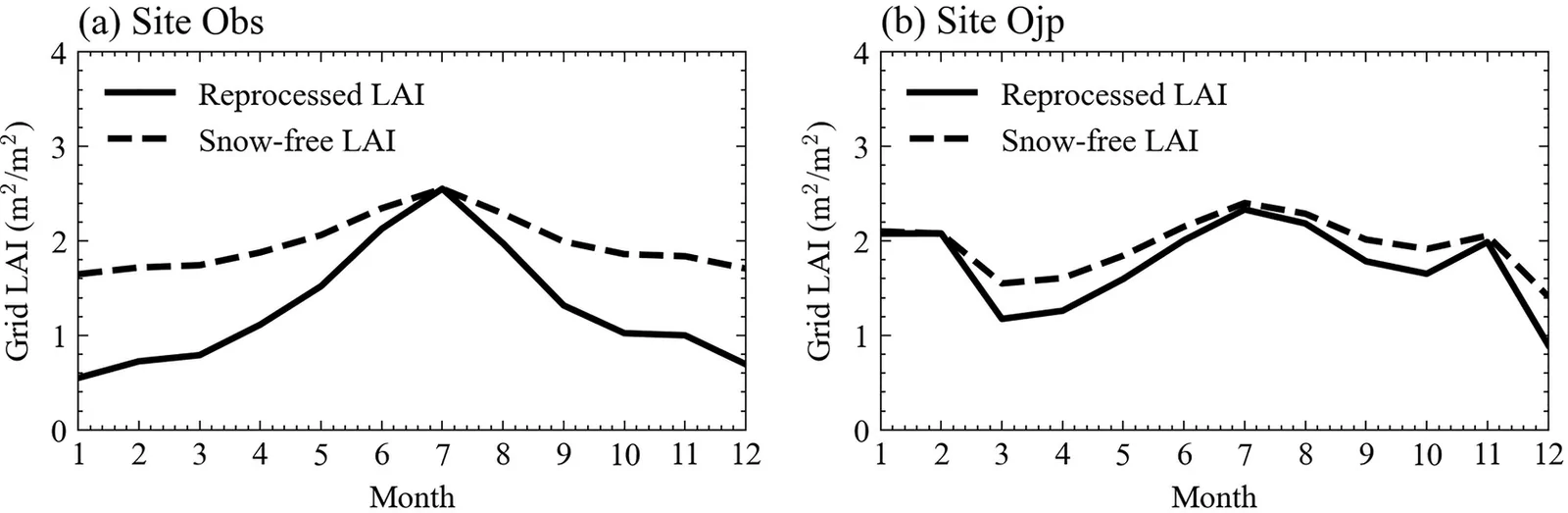
Time series of reprocessed LAI and snow-free LAI at (**a**) Obs and (**b**) Ojp sites.

The two study sites dominated by evergreen needleleaf forest were selected from the Snow Model Intercomparison Project^74^ (SnowMIP). At the Obs site, the pronounced underestimation of winter LAI was effectively alleviated after applying the leaf-lifespan-based constraint (Fig. 7), with the mean winter LAI increasing from 0.65 to 1.69 m^2^/m^2^. At the Ojp site, the reprocessed LAI showed little contrast between winter and summer but exhibited noticeable reductions during the transitional seasons (spring and autumn). These seasonally depressed LAI values were also alleviated after correction, with increases of 0.19, 0.18, and 0.32 m^2^/m^2^ in autumn, winter, and spring, respectively.

**Fig. 8:**
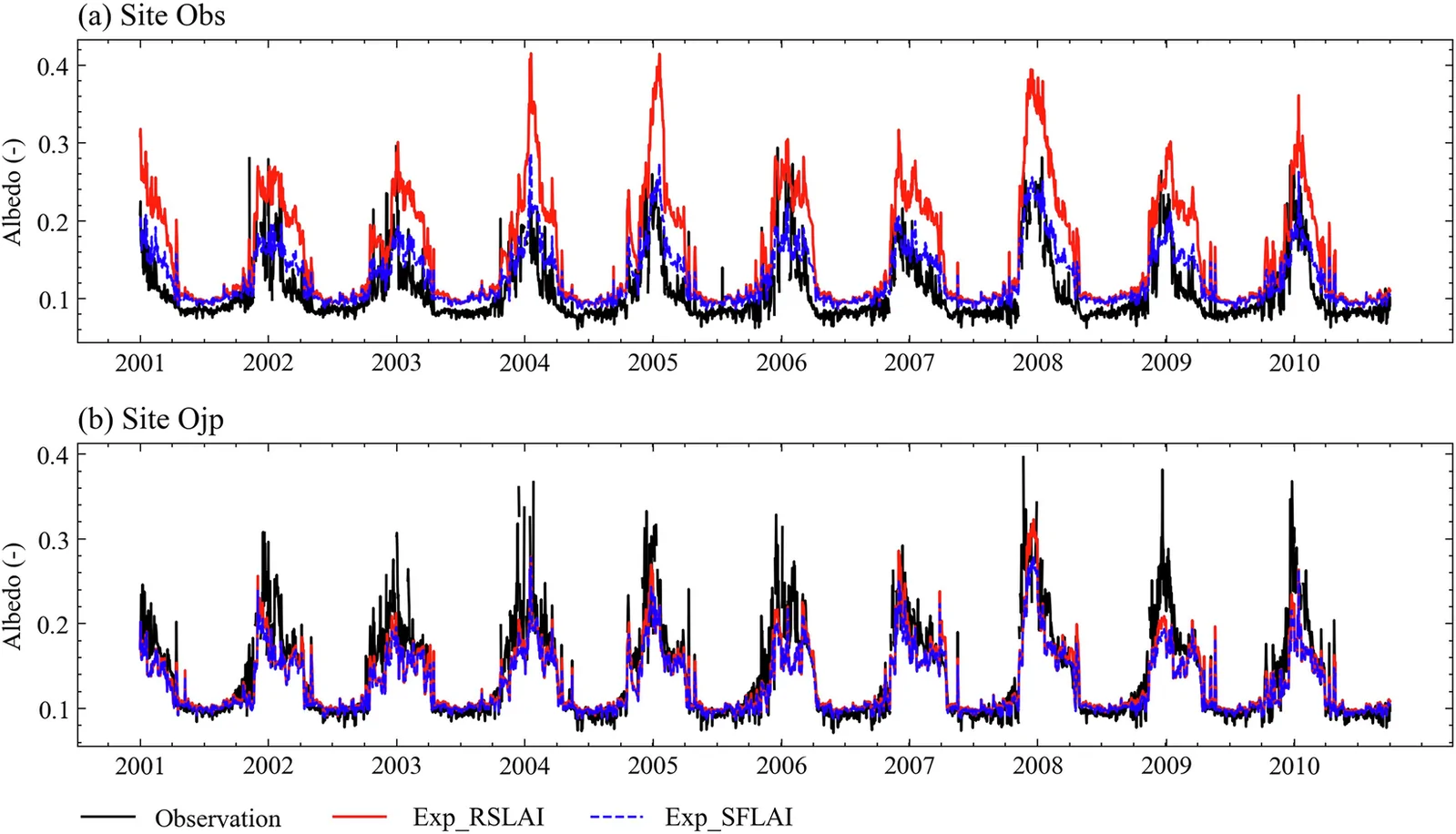
Time series of daily mean albedo from different experiments and observations at (**a**) Obs and (**b**) Ojp sites.

The observed and simulated daily albedo time series for different experiments are shown in Fig. 8. At the Obs site, the Exp_RSLAI experiment, which uses reprocessed LAI, generally captures the observed albedo during the snow accumulation period (Fig. 8a). However, it substantially overestimates albedo during both the peak-albedo period and the snowmelt period, during which the observed values progressively decline. This discrepancy is largely attributable to the low bias in the remotely sensed LAI, whose underestimation implies a larger fraction of snow-covered background and consequently leads to a pronounced high bias in simulated albedo. When the snow-free LAI was applied in Exp_SFLAI, the overestimation during both the peak-albedo stage and the melt phase was substantially reduced. The corrected green leaf area becomes exposed during melting, lowering the background albedo. Consequently, Exp_SFLAI shows much better agreement with the observations, with RMSE decreasing from 0.06 to 0.03 (Table 3).

**Table 3:** Statistical metrics for validating simulated daily mean albedo against observations.

		Exp_RSLAI	Exp_SFLAI
Site Obs	R	0.83	0.84
	MBE	0.05	0.02
	RMSE	0.06	0.03
Site Ojp	R	0.88	0.90
	MBE	−0.01	−0.01
	RMSE	0.03	0.03

R: correlation coefficient; RMSE: root mean square error; MBE: mean bias error (average of simulated minus observed values).

At the Ojp site, both experiments reproduce the observed albedo well, with evaluation metrics indicating even better performance than that at the Obs site (Fig. 8b). Exp_SFLAI shows a slightly higher correlation than Exp_RSLAI, whereas the simulated albedo from the two experiments is similar, which can be attributed to the relatively small differences in LAI between them. The good agreement with observations suggests that, at this site, the reprocessed LAI exhibits smaller intra-annual variability, reflecting the evergreen characteristics of the canopy, and that the model effectively captures the albedo dynamics.

**Fig. 9:**
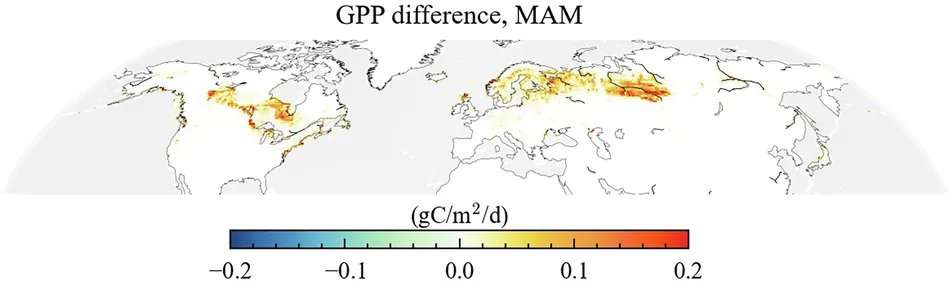
Spatial differences in GPP (Exp_SFLAI − Exp_RSLAI) for the 2001–2003 climatological mean during MAM.

Region-scale simulations were conducted to evaluate the impacts of LAI correction on albedo across the northern mid- to high-latitudes (25–80°N). The simulations covered the period 1996–2003, with the first five years used for spin-up and 2001–2003 selected for analysis to match the observational period of albedo data. As shown in Fig. 9, the application of snow-free LAI exerts a direct influence on GPP, with a pronounced increase in boreal forest regions during spring. This increase in spring GPP primarily reflects the correction of underestimated LAI under snow-covered conditions in conventional satellite products. In boreal forests, evergreen needles are often partially obscured by snow, leading to unrealistically low LAI in winter and early spring. By restoring physically reasonable leaf area, the snow-free LAI improves the simulated canopy light interception at the onset of the growing season, thereby directly enhancing modeled photosynthesis and GPP. This effect is particularly evident in boreal regions, where long snow seasons coincide with evergreen vegetation.

Observational albedo data for validation were derived from the MODIS product^75^ and processed to generate 2001–2003 climatological means^28^, which also serve as the default dataset in the Community Land Model (CLM) diagnostics package. This data is provided on a T42 Gaussian grid, with an effective horizontal resolution of approximately 2.8°. Sixteen-day MODIS albedo observations had been preprocessed to monthly means, retaining only high-quality, cloud- and snow-free data, and missing values for grid cells over the three-year period were filled by linear interpolation from adjacent months.

**Fig. 10:**
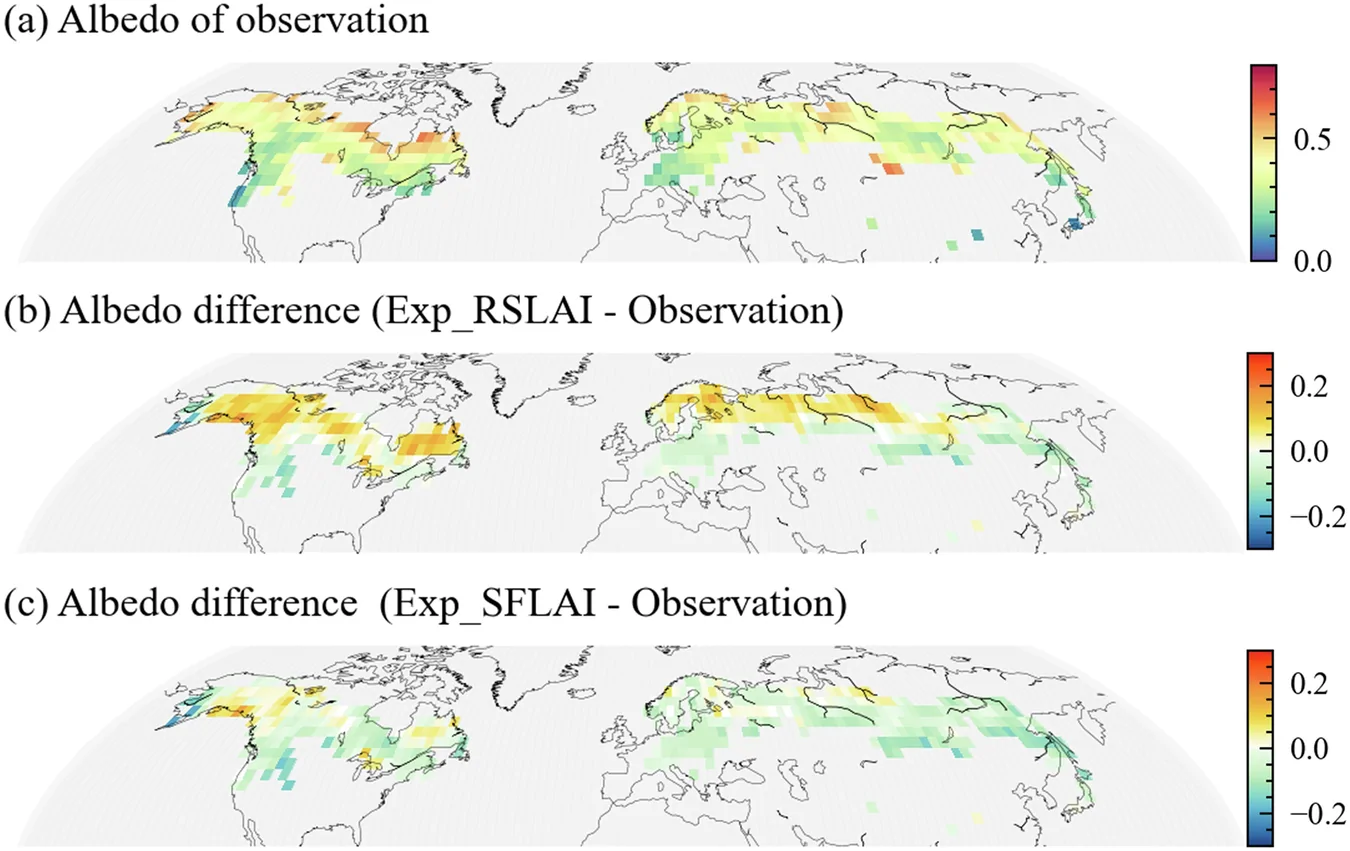
Multi-year mean albedo (2001–2003) from observations and simulations. (**a**) Observed albedo; (**b**) albedo difference between Exp_RSLAI and observations; (**c**) albedo difference between Exp_SFLAI and observations.

The simulated winter albedo exhibits pronounced sensitivity to the LAI data employed. Figure 10 presents the spatial differences between simulated and observed winter albedo for the two experiments, with grid cells masked where the annual mean difference between snow-free LAI and reprocessed LAI exceeds 0.1, thereby highlighting regions where LAI correction is influential. In the observed albedo map, winter albedo generally increases with latitude, reflecting the combined effects of increasing snow cover extent and reduced canopy masking at higher latitudes. When the reprocessed LAI is used, the model systematically overestimates albedo in regions with high observed winter albedo, particularly in snow-dominated areas. This bias can be attributed to the underestimation of LAI in the remote-sensing product, which increases the fractional exposure of snow within the surface background and consequently leads to higher simulated albedo. In contrast, the application of snow-free LAI substantially reduces the positive albedo bias across most high-latitude regions. The improved agreement with observations suggests that LAI correction effectively restores canopy masking over snow-covered surfaces, thereby enhancing the physical realism of winter albedo simulations.

**Fig. 11:**
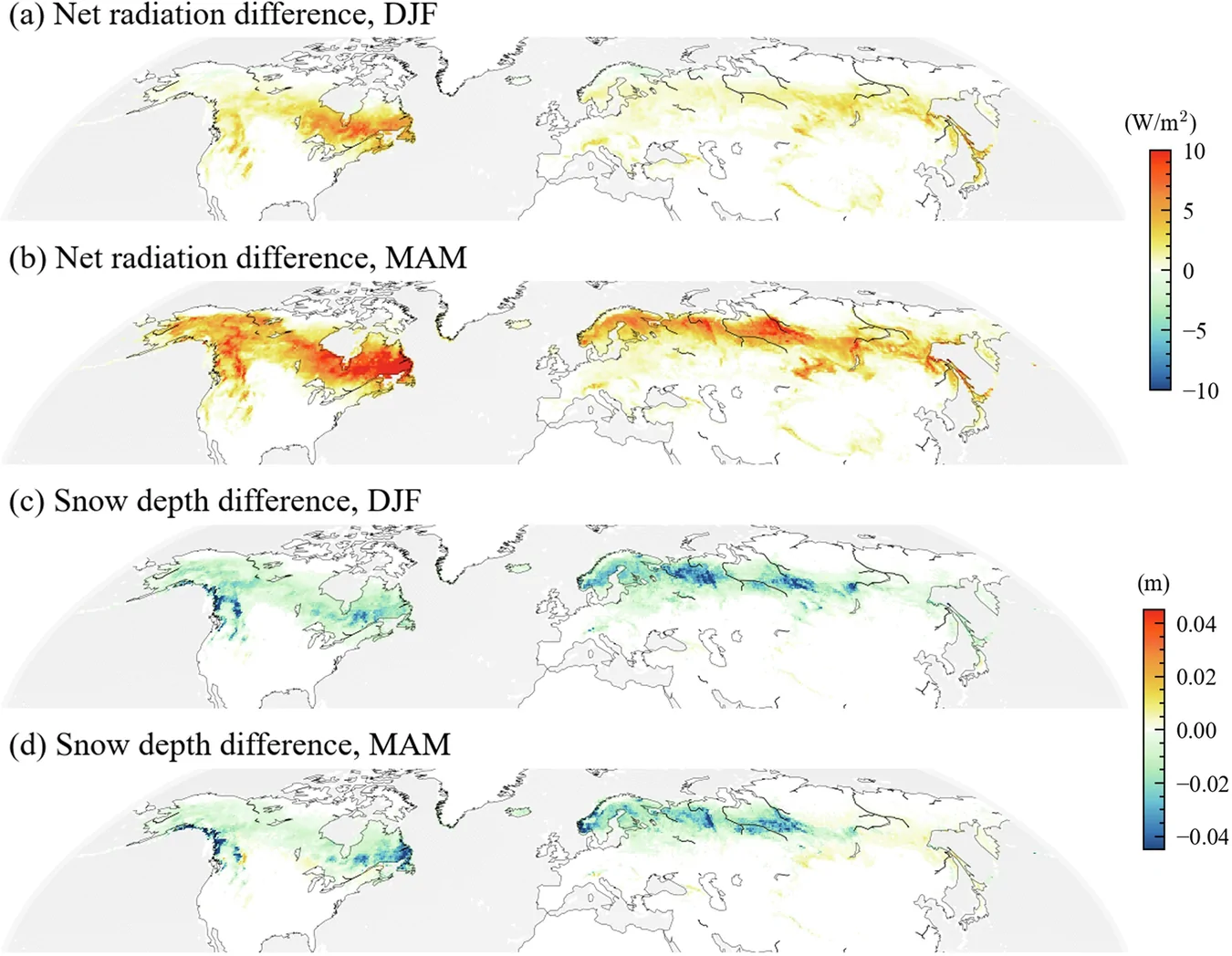
Differences in net radiation (**a,b**) and snow depth (**c,d**) between Exp_SFLAI and Exp_RSLAI for the 2001–2003 climatological mean in DJF and MAM (Exp_SFLAI − Exp_RSLAI).

As albedo decreases due to the application of snow-free LAI, a larger portion of incoming solar radiation is absorbed by the canopy, directly increasing net radiation at the surface (Fig. 11). This effect is more pronounced in spring than in winter because incoming radiation is stronger, despite similar LAI increases in both seasons. In addition, the denser canopy resulting from snow-free LAI intercepts more snowfall, reducing snow accumulation in both winter and spring. These changes not only alter the surface energy balance but also modify snow accumulation and distribution within and above the canopy, providing a more physically realistic representation of snow-vegetation interactions in boreal forests.

## Usage Notes

To facilitate the interpretation and appropriate use of the dataset, several methodological considerations are outlined below. A simple and conservative correction strategy is adopted in this study. The method relies on leaf lifespan to provide a physiological constraint on the minimum LAI of evergreen vegetation, while preserving the temporal structure of the satellite-derived LAI time series. This approach assumes that remote sensing products can reasonably capture the timing of seasonal variations, whereas systematic biases are mainly associated with the magnitude of LAI under snow conditions. Consequently, the correction focuses on adjusting LAI amplitude without reconstructing the full seasonal trajectory.

We note that this method is primarily applicable to evergreen PFTs and does not explicitly differentiate between specific snow cover conditions. Instead, a static snow mask is applied based on whether snow is detected at any time within a year, and the correction is uniformly applied to the entire annual time series. This simplified treatment may, however, introduce limitations under certain conditions. In particular, the correction may lead to overestimation of winter LAI under disturbance events such as severe drought, wildfire, or pest outbreaks, where vegetation remains substantially reduced. The magnitude of this effect depends on the persistence of the disturbance. When vegetation does not recover within the annual cycle, the annual maximum LAI is also reduced, which partially constrains the lower bound in our approach. As a result, the correction is moderated and does not typically lead to unrealistically high values. Nevertheless, this limitation should be considered when applying the dataset in regions affected by prolonged or repeated disturbances.

In addition to the limitations described above, uncertainty arises from the representative leaf lifespan values assigned to each evergreen PFT. The assigned leaf lifespan for each PFT represents a first-order physiological constraint and follows the level of generalization inherent in the PFT classification. It does not explicitly account for spatial variability driven by environmental factors such as climate, nutrient availability, stand age, or disturbance history. Within-PFT variability in leaf lifespan propagates to the estimated lower bound of PFT LAI (${LA}{I}_{\min }^{{\prime} }$) and therefore influences the magnitude of adjustments under snow conditions. To quantify this, we conducted a sensitivity analysis by perturbing leaf lifespan within ± 1 standard deviation of its PFT-level mean, with the lower bound constrained to 1 year to preserve the physiological characteristic of evergreen vegetation. We note that the full PFT LAI time-series is bounded between ${LA}{I}_{\min }^{{\prime} }$ and the satellite-derived maximum (${{LAI}}_{{RS},\max }$), so its sensitivity to leaf lifespan is smaller than that of ${LA}{I}_{\min }^{{\prime} }$ alone. The analysis therefore provides a conservative upper bound on the actual uncertainty in the corrected dataset.

**Fig. 12:**
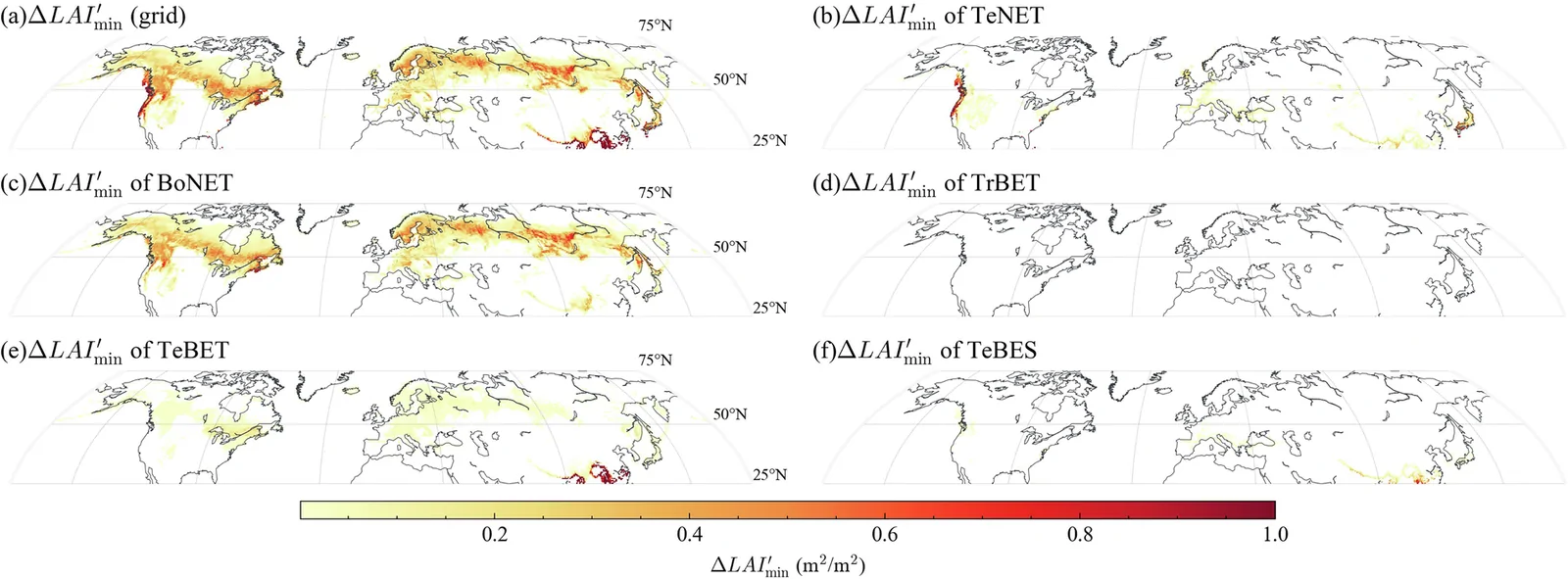
Grid ${{LAI}}_{\min }^{{\prime} }$ sensitivity to leaf lifespan uncertainty (**a**) and contribution from each of the five corrected evergreen PFTs (**b–f**) in snow-affected grids at 0.5° resolution in 2005. Contributions are defined as the product of the per-PFT uncertainty and its fractional coverage within the grid, and sum to the total shown in (**a**). Grids without snow-induced LAI correction are masked.

Figure 12 shows the grid-scale uncertainty in ${LA}{I}_{\min }^{{\prime} }$ and the contribution from each of the five corrected evergreen PFTs, defined as the product of the per-PFT uncertainty and its fractional coverage within the grid cell. The dominant contributor across most snow-affected regions is BoNET, reflecting its extensive overlap with snow-covered areas at mid-to-high latitudes of the Northern Hemisphere. The other three PFTs (TrBET, TeBET, TeNET), despite their higher SD, contribute negligibly owing to their limited presence in snow-affected areas. The area-weighted mean grid-scale uncertainty is 0.22 m^2^/m^2^, and 95% of the snow-affected grids show uncertainty below 0.6 m^2^/m^2^, which is comparable to the typical RMSE of satellite LAI retrievals against ground measurements^32^. Therefore, the uncertainty introduced by the correction is within a generally acceptable range for large-scale model applications.

Direct validation of the snow-free LAI against ground-based measurements during the snow-covered season is currently limited by the scarcity of snow-season canopy LAI observations in publicly accessible monitoring networks. Across the sources we examined, site-level LAI is almost exclusively reported as a growing-season maximum, and ground sampling protocols at major observation networks are generally designed for and conducted within the growing season. Furthermore, accurately measuring LAI in the field during the snow-covered season typically requires destructive sampling, which is labor-intensive and spatially limited. This challenge has similarly constrained prior work: Wang *et al*.^17^ adopted an albedo-based indirect validation strategy for the same reason, which we follow here. We acknowledge the absence of direct validation of LAI during the snow season as a limitation of the current dataset. Addressing this gap will require targeted field campaigns at boreal and temperate evergreen sites to establish the observational benchmarks needed for rigorous evaluation of snow-affected LAI products, including our own.

This dataset provides snow-free LAI, which differs from the remote sensing-derived LAI that only reflects green leaves seen by sensors. It is therefore particularly recommended for land surface model applications involving snow-related processes on vegetation, such as snow interception, unloading, and sublimation. The evaluation of the snow-free LAI dataset in this study is conducted using a single land surface model (CoLM 2024), which explicitly represents vegetation-snow interactions, including canopy snow masking effects. As a result, the simulated impacts of LAI on surface albedo, radiation, snow depth and GPP reflect the sensitivity of this model to vegetation structural changes under snow-covered conditions. Users should be aware that the magnitude of these responses depends on how vegetation-snow interactions are parameterized in land surface models, and models lacking explicit canopy snow masking may exhibit different responses to LAI variations. Therefore, caution is advised when directly extrapolating the quantitative impacts reported in this study to other modeling frameworks.

## Data Availability

The code used for deriving PFT-specific leaf lifespan and generating the snow-free LAI dataset is publicly available at https://doi.org/10.5281/zenodo.19905548.
